# Deadly meningitis outbreak in Rahim Yar Khan: A call for reinforced public health and disease prevention measures in Pakistan

**DOI:** 10.1002/hsr2.2024

**Published:** 2024-04-01

**Authors:** Victor Abiola Adepoju, Safayet Jamil, Mohammad Shahangir Biswas

**Affiliations:** ^1^ Department of HIV and Infectious Diseases Jhpiego (An Affiliate of John Hopkins University) Abuja Nigeria; ^2^ Department of Public Health Daffodil International University Dhaka Bangladesh; ^3^ Department of Biochemistry and Biotechnology Khwaja Yunus Ali University Sirajganj Bangladesh

Dear Editor,

The recent meningitis outbreak in Rahim Yar Khan led to 12 deaths including six children. This event highlights the urgent need for Pakistan's public health system to improve its readiness and efficiency to prevent future outbreaks. Dr. Hassan Khan, CEO of the District Health Authority, noted that after reports of the outbreak, authorities dispatched emergency response teams to the affected area, Mouza Tajpur Pirwala in Ruknpur. This outbreak however is not an isolated incident. According to the Dawn News, meningoencephalitis, a severe form of meningitis, was responsible for the deaths, revealing this as an underlying health issue that must be addressed.^1^ Pathogens such as bacteria, viruses, and fungi can cause meningitis, an inflammation of the protective membranes that cover the brain and spinal cord.^2^ It is a serious and potentially fatal disease. Without prompt diagnosis and effective treatment, meningoencephalitis can lead to serious complications such as brain damage, hearing loss, and learning disabilities. Furthermore, meningitis not only kills people but has a significant economic impact on communities. Among infants and children with suspected meningoencephalitis in the United States, the mean hospitalization cost for infants and children was $12,759 and $11,119, respectively, with medication and laboratory test costs of $834 and $1771 for infants and $825 and $855 for children, respectively.^3^

The economic impact of a large‐scale outbreak can be devastating for communities, especially in low‐ and middle‐income countries like Pakistan.

According to the Borgen Project (2022), the resurgence of polio in Pakistan emphasizes the importance of maintaining strong public health systems, particularly in terms of vaccination and disease prevention.^4^ Meningitis, a vaccine‐preventable disease, is a pressing concern, and the government and health authorities must take the necessary steps to halt its spread and the spread of other vaccine‐preventable illnesses.^2^ A comprehensive and multifaceted approach is required to effectively combat meningitis outbreaks. This includes successfully implementing vaccination programs, improving surveillance systems for early detection and response, and providing appropriate medical care and support to those affected by the disease.^2^

The recent meningitis outbreak in Rahim Yar Khan has highlighted the need for improved public health systems and disease prevention efforts in Pakistan. It is critical that the government and health officials act quickly to prevent future outbreaks by increasing funding for public health programs, increasing vaccination coverage, and providing appropriate medical care and support to those affected. Raising awareness in local communities about the dangers of meningitis and the importance of vaccination is critical. The government and health officials must work closely with these communities to educate them on the disease's signs and symptoms, as well as where to get medical care and support.^5^

The MenAfriVac campaign, New Zealand's comprehensive vaccination program, the UK's detailed prevention plan, and Brazil's public health campaigns demonstrate effective models for combating meningitis, through mass vaccination, targeted interventions, and public education. Examples like these, in contrast to Pakistan's fragmented approach, show that coordinated strategies like these are crucial for the ultimate reduction in disease volumes. Pakistan could better manage its own fight against meningitis, by learning from the models of mass public engagement, targeted vaccination efforts, and grassroots awareness‐building that truly manage to rid nations of the threat. Furthermore, to prevent and control future outbreaks, the government must increase funding for meningitis research to better understand the disease and develop more effective treatments.

Finally, the recent meningitis outbreak in Rahim Yar Khan emphasizes the importance of strengthened public health systems and effective disease prevention measures in Pakistan. To address this issue and prevent future occurrences, the government and health authorities must act immediately. It is critical to provide proper healthcare for citizens and protect them from preventable diseases.

## AUTHOR CONTRIBUTIONS

**Victor Abiola Adepoju**: Conceptualization; methodology; software; investigation; writing—original draft; data curation. **Safayet Jamil**: Conceptualization; methodology; data curation; investigation; writing—original draft. **Mohammad Shahangir Biswas**: Conceptualization; investigation; project administration; supervision; writing—review & editing; methodology.

## CONFLICT OF INTEREST STATEMENT

The authors declare no conflict of interest.

## TRANSPARENCY STATEMENT

The lead author Victor Abiola Adepoju affirms that this manuscript is an honest, accurate, and transparent account of the study being reported; that no important aspects of the study have been omitted; and that any discrepancies from the study as planned (and, if relevant, registered) have been explained.

## Data Availability

Since no new data were created or analyzed in this study, data sharing is not applicable to this article; all of the data underlying the results presented are available in the article.

## References

[1] Jafri Z . Probe confirms meningoencephalitis behind 12 ‘mysterious’ deaths in Rahim Yar Khan. *Dawn News*. 2023. https://www.dawn.com/news/1730142

[2] WHO . Meningitis. World Health Organization; 2020. http://www.epi.gov.pk/vaccine-preventable-diseases/meningitis/

[3] Balada‐Llasat JM , Rosenthal N , Hasbun R , et al. Cost of managing meningitis and encephalitis among infants and children in the United States. Diagn Microbiol Infect Dis. 2019;93(4):349‐354. 10.1016/j.diagmicrobio.2018.10.012 30442508

[4] Borgen Project . Resurgence of polio in Pakistan. The Borgen Project. 2022. https://borgenproject.org/resurgence-of-polio-in-pakistan/

[5] Farooq S . Meningitis outbreak reported in RY Khan. 2023. https://tribune.com.pk/story/2394431/meningitis-outbreak-reported-in-ry-khan

